# Temporal patterns of liver and adipose lipase abundance across the periparturient period in multiparous Holstein dairy cows

**DOI:** 10.3168/jdsc.2024-0613

**Published:** 2024-09-06

**Authors:** S.J. Kendall, H.T. Holdorf, R.S. Pralle, H.M. White

**Affiliations:** 1 Department of Animal and Dairy Sciences, University of Wisconsin–Madison, Madison, WI 53706; 2 School of Agriculture, University of Wisconsin–Platteville, Platteville, WI 53818

## Abstract

Lipases such as patatin-like phospholipase domain-containing protein 3 (PNPLA3) exist in multiple tissue types. In subcutaneous adipose tissue, PNPLA3 was not altered during the periparturient period. Conversely, strong associations between liver PNPLA3 and liver triglyceride content peripartum were identified and confirmed to be causative using knockdown approaches in a primary bovine hepatocyte model. The objective of this research was to characterize adipose and hepatic lipase abundance, as well as abundance of hepatic transcription factors (TF) involved in adipose and liver tissue lipolysis to determine potential impact on bovine fatty liver development and recovery. Adipose and liver tissue biopsy samples were collected from −28 to +56 d relative to calving (DRTC) from multiparous Holstein dairy cows randomly assigned to either a control (CTL) or ketosis induction protocol (KIP) diet. Clinical ketosis (blood BHB ≥ 3.0 m*M*) was used as a cowside indicator of successful induction and was achieved in CTL (n = 2) and KIP (n = 12) cows. Both adipose and liver abhydrolase domain-containing protein 5 and adipose triglyceride lipase (ATGL) differed across DRTC, as did liver perilipin 1. Hepatic mature and immature carbohydrate response element binding protein, and sterol regulatory binding protein 1c, varied or tended to vary across DRTC × treatment. Neither adipose nor liver hormone sensitive lipase (HSL) were responsive to treatment or varied across DRTC, but adipose HSL was positively associated with nonesterified fatty acids at +1 DRTC. Adipose and liver ATGL was associated with liver triglycerides across all DRTC. Patterns of adipose HSL and ATGL abundance did not explain the increase in nonesterified fatty acids observed at +1 DRTC in this study. Future investigation of lipase abundance in other adipose tissue depots is still needed to better explain regulation during this time. Bovine fatty liver development and recovery appears to be contingent upon liver PNPLA3 abundance, rather than liver ATGL.

## Highlights

• Liver adipose triglyceride lipase may support patatin-like phospholipase domain-containing protein 3 to clear liver lipids postpartum.
• Liver mChREBP, iChREBP, and SREBP1c may contribute to lipase regulation.
• No adipose lipase abundance quantified herein was correlated with increased nonesterified fatty acids.

## Graphical Abstract

**Figure:**
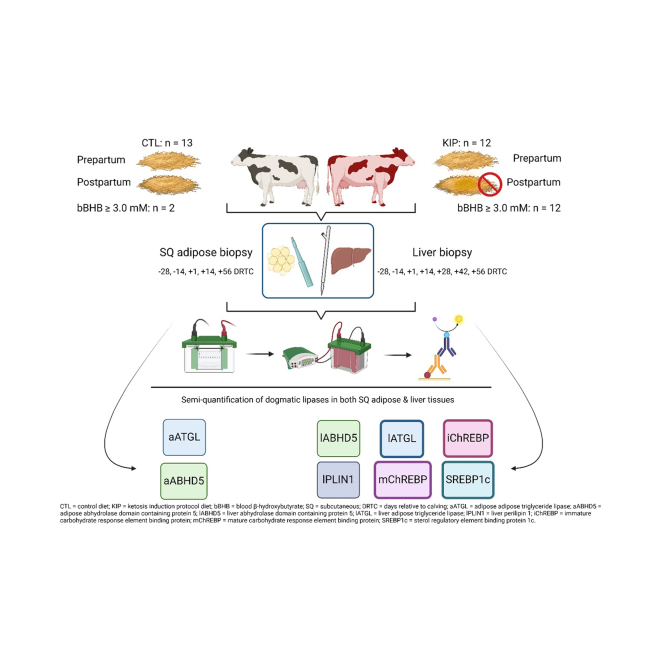
**Summary:** Multiparous Holstein dairy cows were fed according to either a control (CTL) or ketosis induction protocol (KIP; prepartum addition of cracked corn with postpartum feed restriction) diet. Ketosis (blood β-hydroxybutyrate [bBHB] ≥ 3.0 m*M*) was achieved in CTL (n = 2) and KIP (n = 12) cows. Both treatments had subcutaneous (SQ) adipose biopsies taken at -28, -14, +1, +14, and +56 d relative to calving (DRTC), and liver biopsies taken at -28, -14, +1, +14, +28, +42, and +56 DRTC. Protein was isolated, and western blots were created and semiquantified for dogmatic lipases, transcription factors, or both. In adipose, differences in adipose triglyceride lipase (aATGL) and abhydrolase domain-containing protein 5 (aABHD5), and differences in liver by ABHD5 (lABHD5) and perilipin 1 (lPLIN1) were observed by DRTC. A treatment × DRTC effect was observed for liver ATGL (lATGL), immature carbohydrate response element binding protein (iChREBP), mature ChREBP (mChREBP), and sterol element binding protein 1c (SREBP1c). Created with BioRender.com.

Bovine fatty liver disease (**bFLD**) is a metabolic disease that can develop concurrently with ketosis in dairy cows during the periparturient period and occurs in almost half of all periparturient dairy cows (Bobe et al., 2004). Reviews of studies quantifying liver lipid content highlighted associations of high liver triglyceride (**TG**) with reduced yields of milk and milk components (e.g., fat, protein, and lactose), decreased DMI and reproductive performance, and increased incidence of ketosis, hypocalcemia, metritis, and multiple disease occurrences (Jorritsma et al., 2000; Arshad and Santos, 2022). Development and potential recovery of bFLD during the periparturient period is reflective of lipolysis of adipose tissue to provide energy sources, specifically fatty acids (**FA**; Contreras et al., 2017), relative proportion of oxidation, or re-esterification of FA in the liver to either yield energy for cellular use or TG to be stored as lipid droplets within the hepatocytes (Grummer, 1993; Drackley, 1999), lipolysis and subsequent oxidation of transiently stored liver TG (**lvTG**; White, 2020), and packaging and export as very low-density lipoproteins (McFadden et al., 2020).

Although some work in dairy cows has been done to understand the molecular mechanisms behind lipolysis in liver (Pralle et al., 2021; Erb et al., 2022) and subcutaneous (**SQ**) adipose tissues (Koltes and Spurlock, 2011; De Koster et al., 2018), the coordinated response of transcription factors (**TF**) and lipases in these tissues has not been as thoroughly investigated as it has in humans (Alves-Bezerra and Cohen, 2017) and other species. Previous work has identified patatin-like phospholipase domain-containing protein 3 (PNPLA3), a lipase present in liver and SQ adipose tissue, as being a crucial lipase in lvTG clearing during the periparturient period in dairy cows, yet it was unchanged in SQ adipose during the peripartum period (Pralle et al., 2021). To date, no adipose tissue lipase has been noted to increase at the time of calving to explain the increased FA mobilization that is characteristic of this physiological state. Our study objective was to characterize the responsiveness of liver TF (carbohydrate response element binding protein [ChREBP] and sterol regulatory element binding protein 1c [SREBP1c]) and the coordinated response of dogmatic liver and adipose lipases (abhydrolase domain-containing protein 5 [ABHD5], adipose TG lipase [ATGL], hormone sensitive lipase [HSL; total HSL present], phosphorylated HSL [PHSL; active form of HSL], and perilipin 1 [PLIN1]) during the periparturient period in dairy cows and determine their role in bFLD.

Experimental details are described in the companion article (Pralle et al., 2021). Cows were fed either standard peripartum diets or exposed to a ketosis induction protocol (**KIP**). Briefly, pregnant, dry, multiparous Holstein dairy cows (n = 25) were enrolled at −28 expected days relative to calving (**DRTC**; SD ± 4.6 d) and housed individually in tiestalls at the University of Wisconsin–Madison Dairy Cattle Instruction and Research Center (Madison, WI). Cows were randomly assigned to either control (**CTL**; n = 13; ad libitum intake of prepartum [43.70% DM] or lactating cow diet [48.71% DM]) or KIP (n = 12; 6 kg daily top dress of dry, cracked corn [90.2% DM] prepartum; ad libitum intake of dry or lactating cow diet, and then feed restricted to 80% of ad libitum intake at +14 DRTC until the ketosis threshold was reached, thereafter ad libitum intake resumed). All cows enrolled on KIP developed ketosis with the KIP threshold of a blood BHB ≥3.0 m*M*. Given the association between ketosis and bFLD, KIP served as a method to achieve a range of bFLD. Additionally, naturally occurring ketosis was recorded in the CTL group (n = 2) and cows were kept as part of the CTL group; no cows from either group were excluded from downstream analysis. Liver tissue and tailhead SQ adipose tissue biopsies were performed at −28 and −14 (±4.6 d), +1, +14, +28 (liver only), +42 (liver only), and +56 DRTC via blind percutaneous biopsy using a custom-built trocar or via punch biopsy, respectively.

Quantification of protein was completed as described in the companion article (Pralle et al., 2021). Blots fated for ChREBP or HSL probing were biotin blocked using an endogenous biotin blocking kit (E-21390; ThermoFisher Scientific, Waltham, MA) as done previously (Erb et al., 2022) before blocking in 1× Tris-buffered saline + Tween-20 at 0.1% with 5% nonfat milk (Blocking Solution) for 1 h at room temperature (**RT**). Blots fated for all other protein probing were immediately blocked for 1 h at RT with Blocking Solution before probing and washing. All antibodies were diluted in Blocking Solution. Primary and secondary antibodies used are listed for each blot type: liver and adipose blots were probed for ABHD5 (1:1,000 [PA-185666; ThermoFisher Scientific]; 1:5,000 [ab6885; Abcam, Cambridge, MA]) and ATGL (1:500 [ab95322; Abcam]; 1:5,000 [ab6289; Abcam]); liver blots were probed for mature ChREBP (**mChREBP**) and immature (spliced) ChREBP (**iChREBP**; 1:250 [sc-515922; Santa Cruz Biotechnology, Santa Cruz, CA]; 1:5,000 [7076S; Cell Signaling Technology {**CST**}, Danvers, MA]), HSL (1:1,000 [4107S; CST]; 1:2,500 [ab205718; Abcam]), PLIN1 (1:100 [651156; ThermoFisher Scientific]; 1:2,500 [7076S; CST]), and SREBP1c (1:250 [sc-365513; Santa Cruz Biotechnology]; 1:5,000 [7076S; CST]); adipose blots were probed for HSL (1:1,000 [4107S; CST]; 1:5,000 [ab205718; Abcam]), PHSL (Ser563) (1:1,000 [4139S; CST]; 1:5,000 [ab205718; Abcam]), and PLIN1 (1:500 [651156; ThermoFisher Scientific]; 1:5,000 [7076S; CST]). Following Total Lane Protein images, imaging solution (34076; ThermoFisher Scientific) was administered in the dark for 5 min and probed blot images were captured using the ChemiDoc XRS+ Imager and ImageLab 5.0 software (Bio-Rad Laboratories). After imaging, blots were stripped for two 20-min intervals at RT using a buffer (pH 3.0; 200 m*M* glycine, 3.5 m*M* SDS, 10 mL of Tween-20; Abcam) as done previously (Erb et al., 2022). After stripping, blots were rinsed with extra-pure water and Tris-buffered saline, then re-blocked with Blocking Solution for 1 h at RT for the next overnight probe.

Data were checked for normality using PROC UNIVARIATE in SAS 9.4 (SAS Institute Inc., Cary, NC) and root transformed as necessary based on interrogation by Shapiro-Wilk (*P* < 0.05) procedure. Data were analyzed using PROC MIXED (SAS 9.4). Mixed models contained the fixed effect of DRTC, treatment, the interaction, and random effect of cow, block, and cow nested within block. Corresponding −28 DRTC protein abundance was the covariate for each mixed model and were significant (*P* ≤ 0.001) in SQ adipose and liver tissue lipases and TF. Significance was declared at *P* ≤ 0.05 and tendencies at 0.05 < *P* ≤ 0.10. When a fixed effect was significant, means were separated by Tukey–Kramer adjustment. The slice option was used to separate means within the interaction by treatment within a specific DRTC. All data reported are LSM with SEM. Associations between all lipases and TF with nonesterified FA (**NEFA**) and lvTG (on a % DM basis) were explored using raw data using PROC CORR via the Pearson correlation coefficient (SAS 9.4). Figure 2 was generated in R v.4.3.1.

Cow productivity and blood metabolite analysis for CTL and KIP groups are reported in the companion article (Pralle et al., 2021). All cows reported previously were included in the analysis presented herein. For reference, previous results indicated that BW and BCS did not differ between CTL and KIP cows but were affected by DRTC, where BW and BCS were greater prepartum and decreased postpartum. Less negative energy balance was observed for CTL cows on a Mcal basis than KIP cows at wk 4 (+28 DRTC). Milk production tended to be lower in KIP cows than in CTL cows, but increased in both groups over time. Liver TG content differed by DRTC, with greatest lvTG observed at +14 DRTC. Plasma NEFA concentration was affected by a DRTC × treatment interaction.

The primary focus of the companion study was to determine the role of SQ adipose and liver PNPLA3. Investigation in vivo and in vitro indicated that PNPLA3 is a key lipase involved in accumulation and depletion of lvTG in the periparturient period in dairy cows. This lipase differed in abundance in the liver by DRTC, with −14 DRTC having the least abundance (Pralle et al., 2021). In previous work in primary bovine hepatocytes, multiple FA profiles × concentrations inversely affected PNPLA3 abundance (Erb et al., 2022). The focus of the additional investigation here was to determine if other lipases were associated with either lvTG accumulation or depletion, or adipose tissue lipolysis. Given this, the research reported here focused on quantifying liver lipases and TF (Figure 1, panels A–G), exploring associations between both tissue lipases, TF, NEFA, and lvTG (Figure 2, panels A–B), and quantifying SQ adipose lipases (Figure 3, panels A–F).

**Figure 1:**
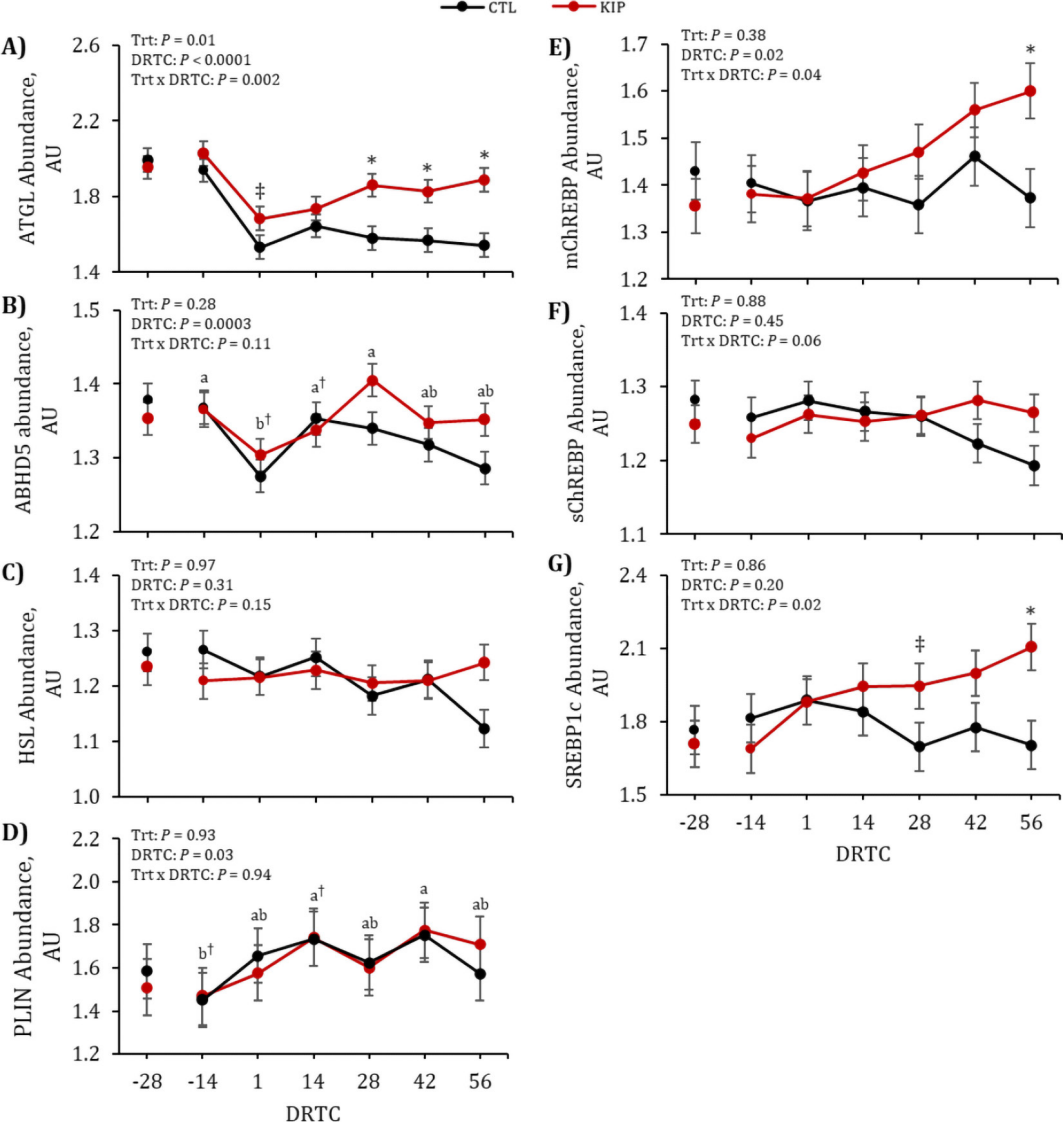
Abundance of hepatic lipases by days relative to calving (DRTC) in control (CTL; black) or ketosis induction protocol (KIP; red) treatments. Data reported are LSM and SEM. Between specific DRTC, letters indicate significant differences via Tukey-Kramer adjustment; daggers (†) indicate tendencies (0.05 < *P* ≤ 0.10) of a letter if a letter is present. In cases of interactions, asterisks (*) indicate a significant (*P* ≤ 0.05) effect and double daggers (‡) indicate a tendency (0.05 < *P* ≤ 0.10) between treatment groups within a specific DRTC from the slice effect. AU = arbitrary unit; Trt = treatment.

**Figure 2:**
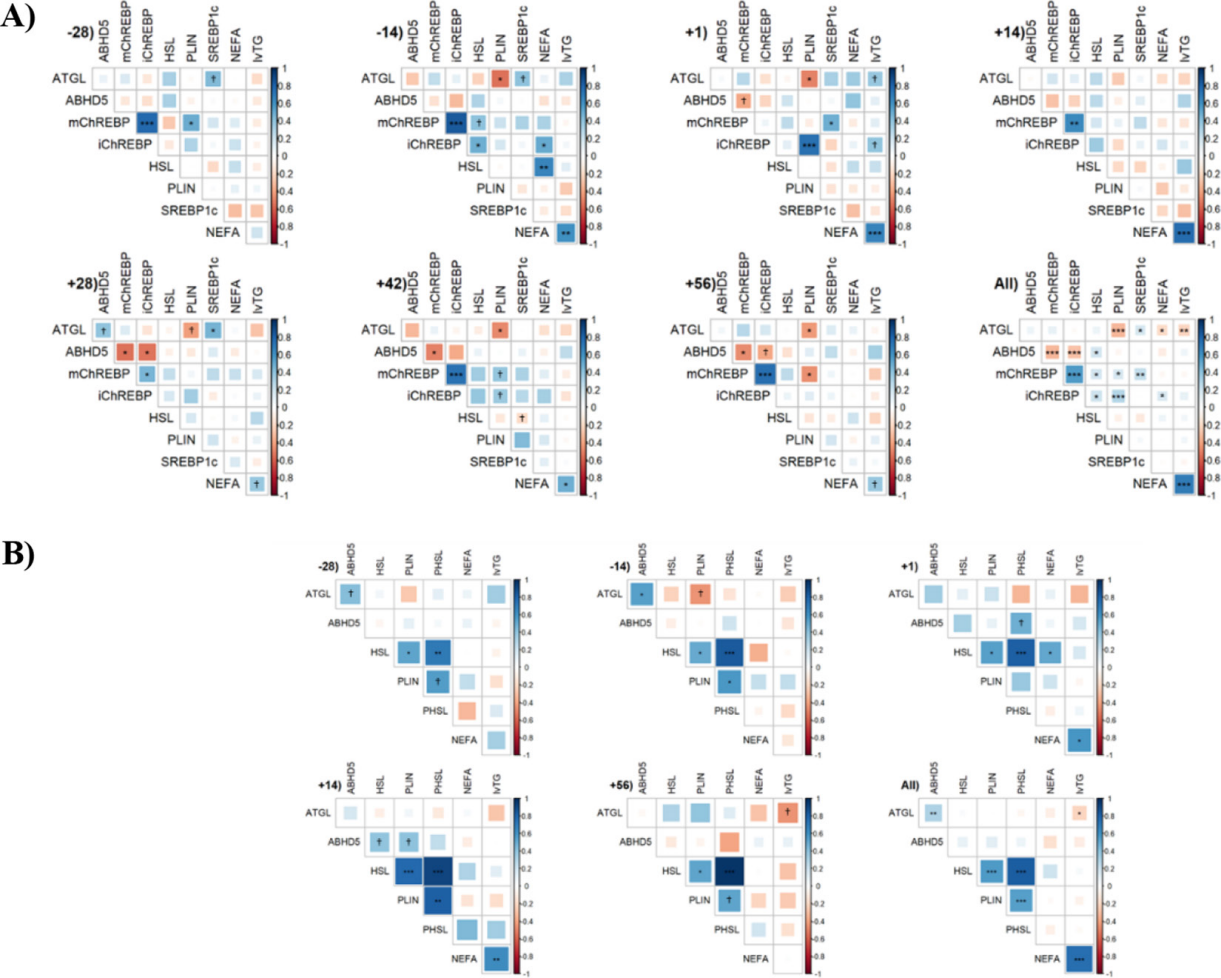
Correlation plots between liver tissue (A) and adipose tissue (B) proteins and circulating NEFA and liver triglyceride (lvTG) content on a % DM basis from −28 through +56 d relative to calving (indicated on each correlation matrix), and across the periparturient period (All). Blue and red indicate positive and negative correlations, respectively; darker and larger squares represent stronger correlations as denoted by the legend. Asterisks (*0.01 < *P* ≤ 0.05; **0.001 < *P* ≤ 0.01; ***P ≤ 0.001) indicate significance and daggers (†0.05 < *P* ≤ 0.10) tendencies.

**Figure 3:**
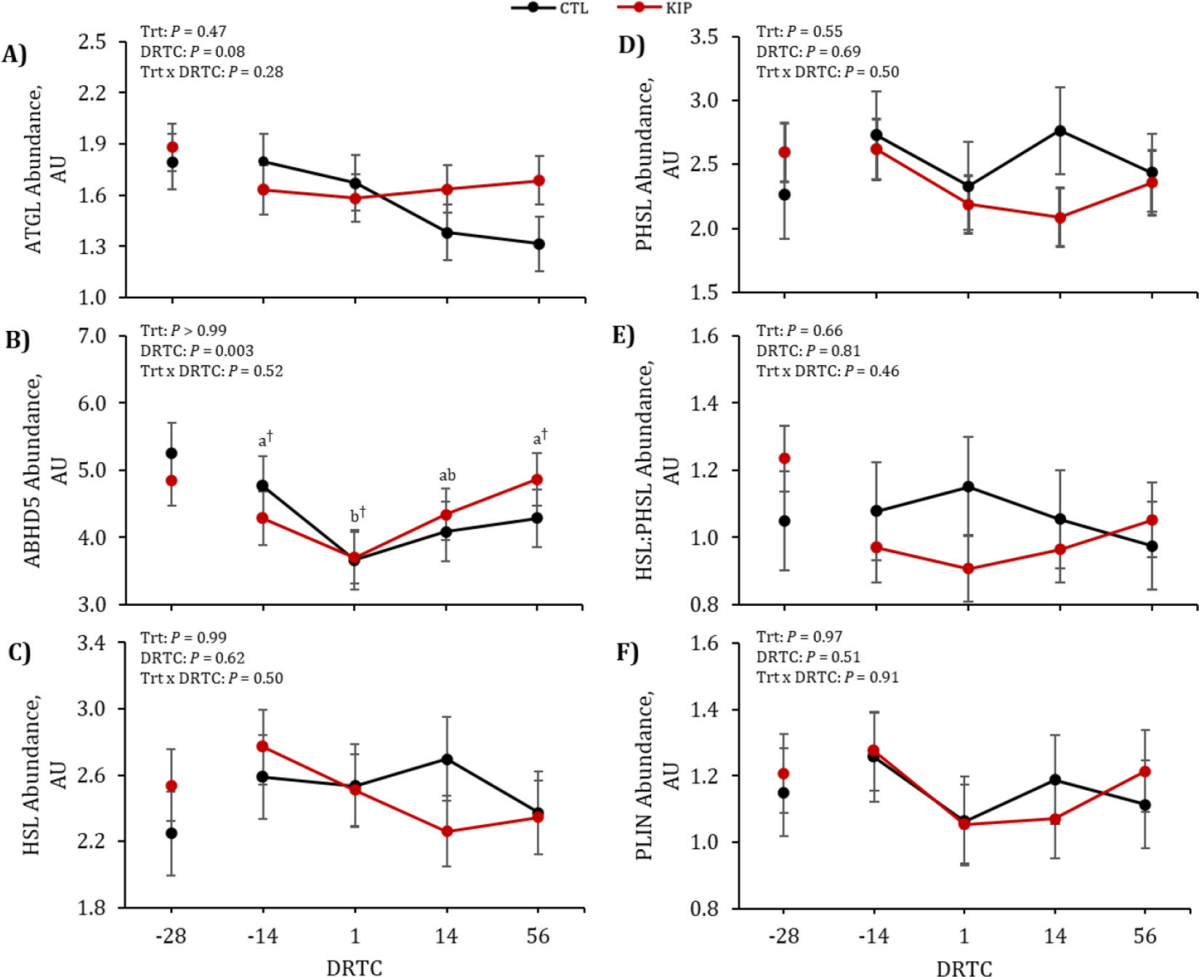
Response of adipose lipase abundances by days relative to calving (DRTC) in control (CTL; black) or ketosis induction protocol (KIP; red) treatments. Data reported are LSM and SEM. Different letters indicate significant (*P* ≤ 0.05) differences via Tukey-Kramer adjustment between specific DRTC. Daggers (†) indicate tendencies (0.05 < *P* ≤ 0.10) of the letter. AU = arbitrary unit; Trt = treatment.

Abundance of liver ATGL had a DRTC × treatment interaction (*P* = 0.002). Liver ATGL abundance tended to be greater (*P* = 0.09) on +1 DRTC in KIP cows and greater (*P* ≤ 0.003) on +28, +42, and +56 DRTC in KIP cows. An association between liver ATGL with NEFA (*P* = 0.03; r = −0.18) and lvTG (*P* = 0.008; r = −0.21) across all days was observed. Specifically, there was a tendency for liver ATGL to be correlated with lvTG at +1 DRTC (*P* = 0.08; r = 0.38). The abundance pattern of ATGL is similar to that of PNPLA3 (Pralle et al., 2021), yet less pronounced across the periparturient period, suggesting that liver ATGL may contribute to postpartum clearing of liver lipids in vivo, working in conjunction with PNPLA3. Despite similar functionality, ATGL does not appear to have compensatory liver lipid clearing postpartum given that cellular TG accumulated and ATGL abundance did not change when PNPLA3 was knocked down (Pralle et al., 2021). Research in other species suggests that ABHD5 is a co-activator to ATGL (Schreiber et al., 2019; Schratter et al., 2022) and interacts with PLIN1 on the lipid droplet (Wang et al., 2008). Abundance of liver ABHD5 differed (*P* = 0.0003) across DRTC. Greater (*P* ≤ 0.003) liver ABHD5 abundance was observed for −14 and +28 DRTC compared with +1 DRTC and tended to be greater (*P* = 0.08) at +14 DRTC than +1 DRTC. A tendency for an association was observed between liver ABHD5 and NEFA (*P* = 0.08; r = 0.40) at +1 DRTC. Although a positive association, this may be due to substrate availability, which is a previously documented relationship between ABHD5, ATGL, and NEFA in other species (Schratter et al., 2022). Abundance patterns of bovine liver ABHD5 and ATGL appeared similar in both treatments, as they do in other species (Schreiber et al., 2019; Schratter et al., 2022).

Abundance of liver HSL was not altered by main effects in this study (*P* ≥ 0.15). This is consistent with findings from an in vitro primary bovine hepatocyte study, where HSL was not altered by treatments (Erb et al., 2022). An association was observed for liver HSL and NEFA (*P* = 0.002; r = 0.65) at −14 DRTC, but no associations between liver HSL and NEFA or lvTG were observed postpartum, suggesting that total HSL is not a rate-limiting lipase in lvTG clearing during bouts of bFLD. Abundance of liver PLIN1 differed (*P* = 0.03) across DRTC: +14 DRTC tended to be greater (*P* = 0.07) and +42 DRTC was greater (*P* = 0.03) in abundance compared with −14 DRTC. Perilipin 1 coats and protects lipid droplets from lipolysis (Wang et al., 2008); therefore, observing that liver PLIN1 tended to be greater at +14 DRTC is in agreement with peak lvTG at this time point in this study (detailed previously; Pralle et al., 2021) and in other studies (Zenobi et al., 2018; Bollatti et al., 2020).

One potential regulatory mechanism for bovine liver lipases could be through TF, which are responsive to various substrates (i.e., NEFA) and regulate pathways with similar functions. For example, ChREBP regulates glycolysis and FA synthesis (Ortega-Prieto and Postic, 2019), and SREBP1c regulates lipogenesis (Iizuka and Horikawa, 2008; Linden et al., 2018). A DRTC × treatment interaction was observed for mChREBP (*P* = 0.04), a tendency for an interaction for iChREBP (*P* = 0.06), and an interaction observed for SREBP1c (*P* = 0.02). Greater mChREBP abundance at +56 DRTC was observed in KIP cows (*P* = 0.01). Similarly, the interaction (*P* = 0.02) for SREBP1c abundance resulted in greater abundance at +56 DRTC (*P* = 0.005) and tended to have greater abundance at +28 DRTC (*P* = 0.07) in KIP cows. An association between iChREBP and NEFA (*P* = 0.05; r = 0.16) at all DRTC was observed, specifically with an association at −14 DRTC (*P* = 0.02; r = 0.51). A tendency for an association between iChREBP and lvTG (*P* = 0.07; r = 0.39) was observed at +1 DRTC. Although there are potential associations regarding iChREBP and lvTG and NEFA found in this study, there is no consistent pattern across the periparturient period. It is unclear why the splice (immature) variant of ChREBP would be more associated with NEFA and lvTG than the mature variant, as the full role of the splice variant remains to be elucidated (Abdul-Wahed et al., 2017).

Understanding the mechanism behind SQ adipose tissue lipolysis may provide insight into bFLD liver lipid accumulation, since FA freed from adipose tissues travels through the bloodstream to the liver and is stored transiently (White, 2020). Abundance of SQ adipose ATGL tended to differ by DRTC (*P* = 0.08), which agrees with previous research (Koltes and Spurlock, 2011). A tendency for association with SQ adipose ATGL and lvTG was observed at +56 DRTC (*P* = 0.07; r = −0.44), and was observed across all DRTC (*P* = 0.04; r = −0.21). These data suggest that ATGL may not be the rate-limiting lipase in SQ adipose tissue. Abundance of SQ adipose ABHD5 was affected by DRTC (*P* = 0.003), where SQ adipose ABHD5 tended to be greater (0.08 ≤ *P* ≤ 0.10) at −14 and +56 DRTC compared with +1 DRTC. Previous research did not note a DRTC effect on SQ adipose ABHD5 (Koltes and Spurlock, 2011); therefore, abundance of SQ adipose ABHD5 may warrant investigation in other sample sets.

Abundance of SQ adipose total HSL, PHSL, HSL:PHSL, and PLIN1 were not affected (*P* ≥ 0.46) by main effects. Similarly, SQ adipose HSL and PLIN1 did not differ across the periparturient period in previous research (Koltes and Spurlock, 2011); however, SQ adipose PHSL differed across DRTC in that study. An association between SQ adipose HSL and NEFA (*P* = 0.04; r = 0.51) at +1 DRTC was observed, along with a tendency for an association across all DRTC (*P* = 0.07; r = 0.21). Prior documentation indicates that phosphorylation of HSL into PHSL activates the enzyme to hydrolyze diglycerides into monoglycerides (Watt et al., 2006; Recazens et al., 2021). Interestingly, SQ adipose PHSL, the active form of HSL, was not associated with NEFA. Associations observed across the periparturient period between SQ adipose HSL and PHSL provide further evidence that these lipases are interrelated in bovine. Similar to this study, previous research noted unchanged SQ adipose PLIN (Koltes and Spurlock, 2011) and unchanged SQ adipose HSL (Koltes and Spurlock, 2011; De Koster et al., 2018). In contrast, other research has noted changed SQ adipose HSL (Locher et al., 2011) and changed SQ adipose PHSL (Koltes and Spurlock, 2011; Locher et al., 2011) abundance across the periparturient period. While most lipase quantification is of protein abundance due to phosphorylation of HSL activating the enzyme (resulting in PHSL) for lipolytic activity to occur, the lipase is regulated both translationally and transcriptionally (Recazens et al., 2021). It must be noted that gene expression and protein abundance do not always exhibit the same pattern for HSL (Recazens et al., 2021), and this is also seen in other enzymes (Erb et al., 2022). Quantification of gene expression of SQ adipose HSL and PLIN1 in vitro identified greater and slightly greater expression, respectively, in energy-restricted cows from −7 through +28 DRTC (Rocco and McNamara, 2013). Although the research presented here does not suggest HSL or PHSL are the rate-limiting enzymes in SQ adipose tissue in these periparturient dairy cows, others suggest HSL (Locher et al., 2011; De Koster et al., 2018) or PHSL (Koltes and Spurlock, 2011) may be. Differences across studies may be attributed to differences in sampling time across the periparturient period, energy balance, and extent of lipolysis. Further work deciphering the differences in HSL gene expression, protein abundance, and phosphorylation in SQ adipose tissue should be conducted to determine the rate-limiting steps.

Although liver and SQ adipose tissue lipases were differentially regulated across the periparturient period, associations observed herein and previously suggest that liver ATGL contributes to, but is not primarily responsible for, lvTG clearing in vivo, and that abundance of liver PNPLA3 was more highly associated with lvTG accumulation or depletion in bFLD (Pralle et al., 2021). More research is warranted to elucidate regulation by the TF examined here and additional TF, including TF such as peroxisome proliferator-activated receptors.

Despite the characteristic increase in NEFA at the time of parturition in numerous studies, a corresponding increase in an SQ adipose lipase responsible for this mobilization is not clear. It is possible that a lipase not yet identified is responsible for the mobilization of NEFA from SQ adipose tissue at parturition, or that mobilized NEFA characteristic of this physiological state are primarily from other adipose depots. Characterizing these lipases in different adipose tissue depots (i.e., visceral, omental, and so on) throughout the periparturient period may provide further insight into metabolic activity of those depots and the contributing proportion of each depot to mobilized energy sources.
